# Space and the parietal cortex

**DOI:** 10.1016/j.tics.2006.10.011

**Published:** 2007-01

**Authors:** Masud Husain, Parashkev Nachev

**Affiliations:** 1 Institute of Cognitive Neuroscience, University College, London, 17 Queen Square, London WC1N 3AR, UK; 2 Imperial College London, Charing Cross Hospital Campus, London W6 8RF, UK

## Abstract

Current views of the parietal cortex have difficulty accommodating the human inferior parietal lobe (IPL) within a simple dorsal versus ventral stream dichotomy. In humans, lesions of the right IPL often lead to syndromes such as hemispatial neglect that are seemingly in accord with the proposal that this region has a crucial role in spatial processing. However, recent imaging and lesion studies have revealed that inferior parietal regions have non-spatial functions, such as in sustaining attention, detecting salient events embedded in a sequence of events and controlling attention over time. Here, we review these findings and show that spatial processes and the visual guidance of action are only part of the repertoire of parietal functions. Although sub-regions in the human superior parietal lobe and intraparietal sulcus contribute to vision-for-action and spatial functions, more inferior parietal regions have distinctly non-spatial attributes that are neither conventionally ‘dorsal’ nor conventionally ‘ventral’ in nature.

## Introduction

Studies in monkeys originally led to the hypothesis that the dorsal visual stream of cortical pathways, extending from the primary visual cortex to the posterior parietal cortex (PPC), has a special role in spatial perception, whereas the ventral stream to the temporal cortex has a key role in object perception [1]. Later, that view was challenged and revised to the proposal that the dorsal stream and PPC have a crucial role in directing visually guided actions [2]. However, it is becoming increasingly apparent that both these models have difficulty in capturing some aspects of human parietal function. In particular, recent findings have begun to question a role for the human inferior parietal lobe (IPL) solely in spatial processes or in the visual guidance of action – ‘vision-for-action’.

Here, we review emerging trends in this field and suggest that a reconsideration of human parietal function is necessary. Close inspection of data from functional imaging and lesion studies reveals that parts of the human IPL are involved in functions that do not involve visually guided action, shifts of spatial attention or spatial memory. Instead, sub-regions within the IPL seem to be involved in the detection of salient new items embedded in a sequence of events and in maintaining or controlling attention over time. We start by considering the current unease that exists over human IPL function.

## Current models of inferior parietal lobe function

In Goodale and Milner's view, the dorsal ‘vision-for-action’ system operates in real time to compute the absolute metrics of the target and its position in the egocentric co-ordinates of the effector (eye or limb) [3]. Thus, according to this scheme, the dorsal stream delivers information direct to the motor system for immediate use for reaching, grasping or eye movements. By contrast, the ventral stream is dedicated to ‘vision-for-perception’, but might have a role in movement planning based on memory of the object and its relationship to other items.

**Figure 1:**
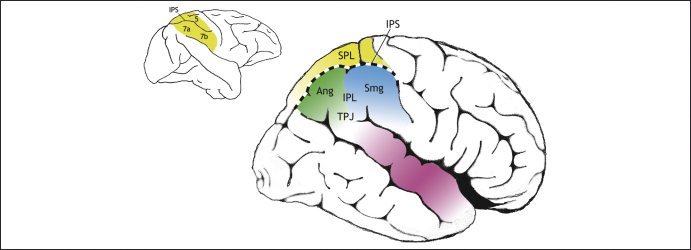
Posterior parietal cortex of macaque monkey (left) and human (right). The human posterior parietal cortex (PPC) is divided by the intraparietal sulcus (IPS) into two parts: the superior parietal lobe (SPL) and the inferior parietal lobe (IPL). The IPL consists of the angular gyrus (Ang) and supramarginal gyrus (Smg) and borders the superior temporal gyrus (purple) at a region that is often referred to as the temporoparietal junction (TPJ). In macaques, the PPC also consists of an SPL (area 5) and an IPL (areas 7a and 7b) but, according to Brodmann [71], the homologues of these macaque regions are all confined to the human SPL (yellow), so the IPL in humans consists of novel cortical areas. Subsequent anatomists such as Bailey and von Bonin [72] disagreed with this scheme, considering the IPL to be similar across both species. It remains to be established whether there are new functional sub-regions within the human IPL.

Such a distinction between dorsal and ventral systems is supported particularly clearly by the results of lesions of the human superior parietal lobe (SPL; Figure 1), which often lead to optic ataxia or misreaching to visual targets [2,4]. The regions of the PPC that have proven far more difficult to fit into any dorsal–ventral dichotomy are the IPL and nearby temporoparietal junction (TPJ). Milner and Goodale acknowledged this from the outset; they speculated that these regions might be a high-level representational system fed largely by the ventral stream and subserving perceptual awareness [2,4]. Their proposal would be consistent with some aspects of hemispatial neglect, a syndrome that often follows lesions of the IPL or TPJ, particularly in the right hemisphere [5,6]. Individuals with neglect often fail to be aware of contralesional objects (to the left for individuals with right parietal damage), even when given unlimited time to explore the world around them.

A different perspective on the IPL has been offered by the scheme proposed by Rizzolatti and Matelli, who suggest that it might be better to consider the superior and inferior parts of the PPC as belonging to two different streams [7]. According to this model, the SPL is part of a distinct ‘dorso-dorsal’ system dedicated to the online control of action, whereas the IPL is part of the ‘ventro-dorsal’ stream that is necessary for action understanding and spatial perception. This model originated from structural and functional considerations of the macaque brain but it has also been applied to the human PPC. In fact, it is possible that in the human brain, the ventro-dorsal system is different in the two hemispheres because deficits of action control (in the form of ideomotor apraxia) are more prominent after left-hemisphere lesions, whereas deficits such as hemispatial neglect are more frequent and severe after right parietal damage.

A third recent view of the PPC also makes a distinction between the SPL and more ventral regions in the PPC, specifically the TPJ, but this hypothesis concerns a dichotomy that incorporates visual-attention functions more directly. Corbetta and Shulman [8] argue that the SPL and parts of the intraparietal sulcus (IPS) have a role in directing visual attention ‘top-down’ to locations or objects in the scene and in selecting responses of effectors (eye or limb). By contrast, the right TPJ acts ‘as a circuit breaker’, for example, by reorienting spatial attention when it has been deployed incorrectly [8]. All these models provide important syntheses of a large body of data that has emerged from studies of monkey and human PPC, but there are other findings, which we consider later, that are still not easily incorporated into these frameworks.

## Functional imaging reveals non-spatial functions

**Figure 2:**
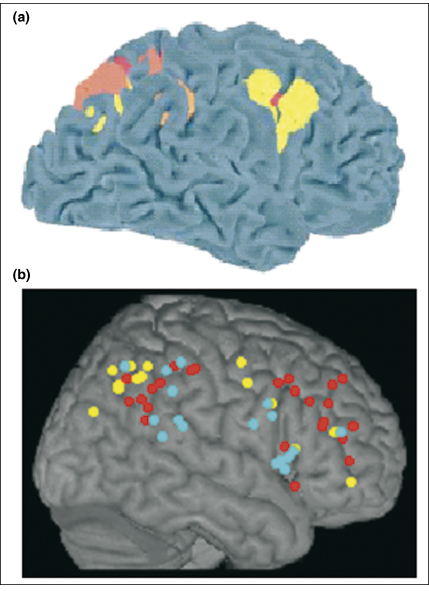
Functional imaging studies. **(a)** Meta-analysis of activations associated with spatial shifts of attention in healthy individuals demonstrate activations in the superior parietal lobe (SPL) and intraparietal sulcus (IPS), plus dorsolateral frontal regions (different colours correspond to findings from different studies). Similar regions are also activated when participants perform spatial working memory tasks. Adapted, with permission, from Ref. [8], © (2002) MacMillan Publishers Ltd. **(b)** By contrast, regions in the inferior parietal lobe (IPL) and IPS, together with more ventral frontal regions, are activated by salient events (cyan), sustained attention (red) and non-spatial selective attention protocols (yellow) such as the attentional-blink paradigm. Adapted from Ref. [18].

Neuroimaging investigations in humans have consistently demonstrated that parts of the SPL or IPS are activated by tasks such as shifting spatial attention, engaging spatial working memory, making saccadic eye movements or reaching to a visual target [8–13] (Figure 2a). In addition, more recent reports have demonstrated evidence in SPL and IPS for topographical representations of contralateral space for saccades to remembered locations [14,15] or in remapping spatial locations across saccades [16,17]. Note that the SPL and IPS activations are often associated with simultaneous activity in the dorsolateral frontal lobe (Figure 2a), in accord with the view that the SPL is part of a dorsal frontoparietal system for directing spatial attention or action. Thus these neuroimaging findings regarding the SPL and parts of the IPS would be consistent with many aspects of current models of PPC function.

However, the results of several other functional imaging studies relating to the IPL and TPJ are very different. They demonstrate that these areas – and more ventral frontal regions – are consistently activated when healthy individuals perform non-spatial tasks [18] (Figure 2b). They have been identified to be active when subjects maintain vigilant attention [9,19–22] or when they are presented infrequently with unusual, salient stimuli in a repetitive stream, as in the ‘oddball’ paradigm [23–26], even when no spatial shifts of attention, eye or limb movements are required. Moreover, these effects occur in visual, auditory or somatosensory tasks and are therefore not modality specific. Some parietal areas in or near to the IPS, together with frontal regions, are also activated when healthy subjects perform non-spatial, selective attention tasks [27–30] such as the attentional-blink protocol, a paradigm that allows measurement of the dynamic capacity of visual attention when stimuli are presented sequentially at one spatial location. Thus regions in the IPL, TPJ and parts of the IPS form a ventral frontoparietal circuit that seems very different from the dorsal one that is active in spatial perceptual, attentional, mnemonic or action tasks (Figure 2b).

These neuroimaging findings suggest both spatial and non-spatial functions are represented in the human PPC, but with a gradient extending from SPL through IPS to IPL of spatial to non-spatial functions [18] and some intermediate regions in the IPS showing activation on both spatial and non-spatial tasks [28]. Corbetta and Shulman's model proposes a slightly different dichotomy between superior and inferior parietal structures, with the TPJ involved in directing attention to salient events, for example, when observers have to redeploy attention to an unexpected peripheral stimulus [8,31]. However, in our view, the IPL and TPJ also play a more general, non-spatial role in sustaining attention over time [18] that is not captured by the Corbetta and Shulman model. New data from transcranial magnetic stimulation also support the view that this region might have a role in non-spatial encoding of salient stimuli [32], as do a range of findings from lesions of the human PPC, which we discuss next.

## Hemispatial, spatial and non-spatial deficits

It has often been suggested that some components of neglect syndrome, the characteristic clinical disorder following right IPL damage, might best be understood using a spatial conceptual framework; for example, a lack of space exploration to one side of the body midline, to one side of the head axis or to one side of the direction of gaze. In fact, there is evidence for all three egocentric spatial reference frames modulating the extent of neglect [33]. But the disorder need not be strictly hemispatial – that is, neglected and non-neglected space might not be demarcated by an abrupt plane that transects space in any egocentric coordinate frame.

Kinsbourne suggested instead that each hemisphere orients attention towards the opposite side of space, with a stronger ‘vector’ exerted by left-hemisphere systems [34]. Hence a strong rightward directional bias would be expected after unilateral right parietal damage (because of unopposed left-hemisphere activity). The model predicts a gradient, rather than a sharp cut-off, in the distribution of visual attention from right to left – a proposal supported by findings of a gradient of response times to stimuli presented at different spatial locations in neglect patients [35]. This model also shows that the unilateral nature of neglect might emerge from a directional bias in just one component of the syndrome; other components that contribute to neglect need not be directionally lateralized.

The key feature of Kinsbourne's directional theory is that it predicts hyperattention (i.e. better performance) to the right. By contrast, the hypothesis advanced by Heilman *et al.* is slightly different, suggesting that there might be some deficits on the right (‘good’) in addition to the left (neglected) side of space [36]. The authors proposed that, although the right PPC might normally direct attention to both sides of space, the left PPC directs attention only to the right hemispace. One implication of this model is that right PPC damage might be associated with not only a severe deficit for items to the left, but also a milder deficit for stimuli to the right [37]. Recent studies show that there are indeed deficits on the right side of space, but they are not necessarily milder than on the left.

Firstly, Duncan *et al.* have demonstrated reduced visual processing and short-term memory capacity, which can be equivalent in severity in left and right hemifields, in IPL patients who have varying degrees of neglect [38]. Importantly, a subsequent study of non-neglect patients revealed that IPL- and TPJ-lesioned individuals were more severely affected than those with SPL involvement [39]. Secondly, processing of visual information from the right visual field – but not the left – might be unselective, with information that is irrelevant to the task being inappropriately prioritized in neglect patients [40]. Thirdly, attention to transient onsets and offsets of visual stimuli is disrupted bilaterally in right IPL patients [41]. Fourthly, detection of briefly presented stimuli is impaired in both visual hemifields, but worse to the left [42]. Finally, spatial functions such as keeping track of object locations over intervening saccades or awareness of changes in their location might all be severely disrupted in patients with right-IPL damage, even on the right side of space [43–46]. All these studies show that damage to the right IPL might lead to deficits that are not confined to one hemispace.

Other investigations have revealed that parietal damage can lead to deficits on non-spatial tasks even when stimuli are presented at only one spatial location. The attentional-blink protocol, which we discussed previously in the context of functional imaging studies, has also been employed in patients to index the dynamic control of visual attention when stimuli are presented sequentially at fixation [47,48]. These investigations have demonstrated that individuals with IPL lesions – including those with spatial neglect – are dramatically impaired in visual-processing ability, even when attention does not have to be shifted across space. By contrast, lesions of the SPL do not lead to attentional-blink deficits [48]. A second series of studies has focused on tests of the ability to maintain vigilant attention. Resection of the right IPL leads to impairment in the ability to sustain visual attention over prolonged intervals [49]. In addition, right-hemisphere neglect patients who have parietal involvement are also impaired at maintaining vigilant attention on non-spatial tasks, regardless of whether the stimuli are auditory or visual [50,51].

This wide range of findings makes it difficult to sustain a simple ‘spatial’ or indeed ‘hemispatial’ account for neglect. Instead, these results reveal that deficits occur on both sides of space in the syndrome; some of them might be spatial in nature, but evidently others are not. The data suggest that a combination of spatial and non-spatial impairments exists in neglect [18], and this might explain why it has been difficult to frame the syndrome in terms of any dorsal–ventral dichotomy (discussed in Ref. [4]).

## Human and monkey parietal cortex might not be the same

Our review of the lesion and imaging data in humans suggests the SPL might have a key role in spatial functions and vision-for-action. We consider this part of the human parietal cortex to have strong similarities to the monkey PPC. By contrast, some regions within the human IPL have non-spatial functions that do not map easily to the ‘dorsal’ stream (see also Refs [2,4]). In fact, monkey parietal cortex might not be a complete model for the human PPC. In our opinion and that of Milner [52], there is no good evidence for a long-lasting and severe neglect syndrome in monkeys such as that seen in humans [53]. Although parietal-cortex or white-matter damage in macaque monkeys leads to various impairments in contralateral space, there is no description of their everyday behaviour, in our view, that equals the profound deficits observed in humans by clinicians. Crucially, there is also no evidence for hemispheric differences for neglect-like symptoms in any monkey model, whereas in humans neglect is far more common and prominent after right-sided damage.

**Figure 3:**
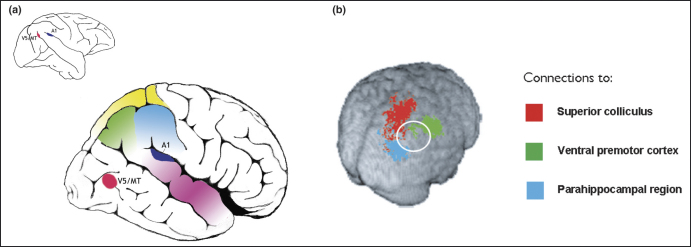
Expansion of posterior brain regions. Human posterior brain regions in the parietotemporal regions have expanded considerably. **(a)** Comparison of the relative positions in macaque (left) and human (right) brains of landmark regions such as primary auditory cortex (A1) and the motion-sensitive area V5/MT reveals how the latter has shifted posteriorly and inferiorly in humans compared with its location in the depths of the superior temporal sulcus in macaque. **(b)** One way to map homologous regions in monkey and human brains is to compare connectivity of regions. The recent study by Rushworth *et al*. [59] demonstrates connections in the human parietal cortex from the superior colliculus (connected to area LIP within the IPS of macaque), ventral premotor cortex (connected to area 7b in macaque) and the parahippocampal region (connected to area 7a in macaque). But there is a region within the human IPL (marked within the white circle) that seems not to have connections to any of these regions and might be a candidate zone for a novel cortical region within the IPL. Panel b adapted from Ref. [59], with permission from Oxford University Press.

Second, although functional imaging, electrophysiological and tractography studies now point to several homologous regions across species [10,13,54,55], there are, in addition, regions of human PPC that seem not to have clear homologues in macaques [55,56]. Comparative anatomy considerations suggest that the IPL has expanded greatly in humans compared with monkeys [57], particularly its posterior aspects – the angular gyrus and TPJ [55]. Comparison of the relative difference in location between functionally homologous regions (e.g. the motion sensitive area V5/MT and primary auditory cortex) reveal a large expansion of the cortex between these areas in humans (Figure 3). The crucial question is whether this expansion is due to the evolution of new areas or simply duplication or enlargement of old ones [57,58].

To answer this question definitively will require converging evidence using several techniques, including perhaps comparative functional imaging. Two other approaches also show considerable promise. First, diffusion-weighted MRI tractography potentially allows homologies to be identified between parietal areas in human and monkey brain that show similar patterns of connectivity. Intriguingly, one recent innovative tractography study demonstrated that a portion of the IPL in humans might not have a clear homology to parts of the PPC in macaque monkeys [59], raising the possibility that this region might be a ‘new’ cortical zone in the human brain (Figure 3). The second method involves combining post-mortem structural imaging with the painstaking process of detailed cytoarchitectonic mapping. This technique has been used to identify a zone in the human posterior IPL that again seems not to have a direct homologue in the macaque brain [60].

## Neither dorsal nor ventral: the human IPL

The findings discussed here raise several important issues. Both the imaging and lesion data suggest that the human SPL, together with parts of the adjoining IPS, have close homologies to the macaque parietal cortex. Thus these areas might be the equivalent of the target of a dorsal stream in monkeys. However, parts of the human IPL seem to be neither ‘dorsal’ nor ‘ventral’. They have non-spatial functions that are not related to object processing, as found in ‘ventral’ stream temporal cortical areas. Instead, they seem to have a role in detecting salient new items embedded in a sequence of events (as indexed by the ‘oddball task’) [23–26] and maintaining or controlling attention over time (as measured by vigilance and non-spatial attentional paradigms) [9,19–22,27–30].

This perspective of human parietal cortex also has important implications for understanding the neglect syndrome [18]. Recent studies of the anatomy of neglect have been controversial (Box 1). Although the argument has often focussed on the contribution of single brain regions it is evident that most patients have large lesions that extend over several crucial areas. Moreover, the syndrome is heterogeneous, with different patients having different combinations of cognitive deficit [51]. Many individuals with neglect have brain damage that spans the cortex of the SPL, IPS and IPL [6,51,61] and the underlying white matter [62]. Given what we know of the functions of these parietal regions, one would expect such damage to lead to a combination of spatial and non-spatial deficits in neglect patients [18], and this is exactly what has been found with careful testing using a battery of spatial and non-spatial tasks [51].

New experimental approaches to the neglect syndrome, using a combination of high-resolution anatomy and behavioural tests designed to assess spatial or non-spatial functions, have begun to show how the heterogeneity in the syndrome might be explained in this way. For example, those neglect patients who have posterior damage and a deficit in keeping track of spatial locations across saccades have lesions that include a small zone in the IPS [43]. By contrast, neglect patients in which the posterior cortex is lesioned but this small area of the IPS is spared do not show this deficit. Similarly, lesions of the right TPJ – but not the SPL – lead to impairments in responding to rare, salient events such as when a target is presented at an unexpected location [63], whereas damage near the IPS is associated with interference from right-sided irrelevant distractors [61]. The interaction of spatial and non-spatial factors, and the precise combination of these, might be crucial in determining the manifestations of neglect across different patients [18].

## Concluding remarks

Current models of the parietal cortex have difficulties in capturing elements of human IPL function. Our review points to the conclusion that parts of the human IPL do not fit a role in spatial processing or vision-for-action, as might be assumed from considerations of the monkey PPC. Rather, some regions in the human IPL seem to participate in the detection of salient new items embedded in a rapid sequence of events and in maintaining or controlling attention over time. This view of parietal cortex can also accommodate findings that demonstrate both homologous and non-homologous sub-regions of parietal cortex in macaque monkeys and humans (Figure 3). Monkeys with parietal lesions might not demonstrate the full-blown neglect syndrome observed in humans because they do not have the complement of spatial and non-spatial deficits that are common following human lesions [18].

In addition, hemispheric differences between left and right IPL are an important feature of the human parietal cortex. Whereas right IPL and TPJ lesions commonly lead to neglect, damage to the homologous region in the left hemisphere in humans often leads to the syndrome of apraxia [64]. However, some left IPL patients might also show evidence of right-sided neglect. Clearly, the precise distinctions in function between left and right IPL remain to be fully established (see Box 2 for Questions for future research). But it is evident that such substantial differences in function across the hemispheres has not been observed in monkeys, demonstrating the need to be cautious when making extrapolations from monkey studies to human parietal cortex.

## Figures and Tables

**Figure I:**
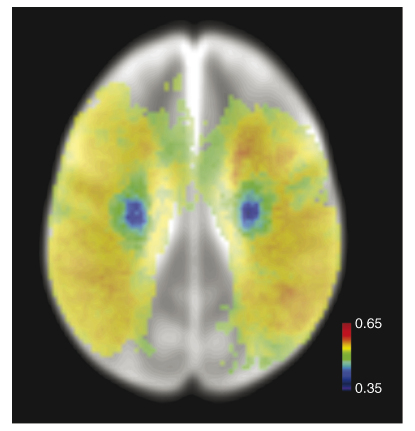
Spatial inhomogeneity of mean lesion volume. Voxel-wise map of the rank correlation between total lesion volume and probability of damage, given a clinical and radiological diagnosis of stroke, derived from a sample of 456 patients. Only voxels affected in eight or more patients are shown. Note the strong centrifugal gradient of mean lesion size, with more peripheral (cortical) voxels being involved in larger lesions. Scale gives value of Spearman's rho.

## Box 1 Anatomy of neglect

The anatomical locus of neglect has recently become the subject of controversy. The debate has centred on evidence derived from patients with focal brain lesions, usually caused by stroke in the territory of the middle cerebral artery (MCA). The region most consistently implicated is the right IPL [5,6]. Recently, an alternative crucial locus has been proposed in the superior temporal gyrus (STG) [65,66]. The disparity between this new proposal and the results of other studies has attracted considerable debate and has been attributed to differences in clinical selection criteria, quality of imaging and the methods of ensuring comparisons between homologous structures across subjects. However, the size of the difference between studies is too large to be accounted for by these factors alone.

It is probable that a single locus for neglect is implausible. The syndrome is functionally heterogeneous, incorporating several component deficits [18,33,51] that are likely to result from disruption of circuits distributed beyond the parietal lobe. Indeed, neglect might occur following damage to lateral frontal regions, consistent with the known frontoparietal circuits revealed by functional imaging in healthy individuals (Figure 2). The syndrome also follows lesions to subcortical structures and even in patients with strokes in the distribution of the posterior cerebral artery (PCA) who do not have direct involvement of lateral parietal or frontal regions [6].

A contributory factor in such cases might be the interruption in functional connectivity between regions: such diaschetic effects are suggested by the correlation between neglect and focal cortical hypoperfusion following subcortical lesions [67]. More directly, damage to frontoparietal fibres has also been shown to be associated with neglect [62,68] whereas lesions affecting the splenium or connections from the parahippocampal region to the parietal cortex have been shown to be predictive of neglect following PCA stroke [6,69,70]. Patients who have chronic neglect are more likely to have involvement of multiple cortical areas, which reinforces this view.

The anatomy of neglect revealed by these studies suggests a complexity that is hard to capture adequately by current lesion-mapping techniques. Most methods rely on performing a statistical test independently at each inter-subject aligned voxel, without any attempt to capture correlations across different brain regions. Because the structure of the vascular tree introduces a marked inhomogeneity in the shape and distribution of stroke lesions, this approach is inherently insecure. Indeed, the only global feature of the lesion commonly modelled – overall lesion size – will inevitably distort any spatial inference because of the strong inhomogeneity in lesion volume across the brain (Figure I). Further progress in this field will have to await the introduction of novel multivariate methods.

However, the investigation of functions that might have no counterpart in non-human primates presents a special challenge. In the absence of detailed neurophysiology and experimental lesion studies, there is little to constrain speculation based on the abundant – yet purely correlational – data generated by functional imaging. It is, therefore, imperative that lesion-mapping methods are developed that match the ease and flexibility of functional imaging and can be widely used to test the predictions generated from it. Only by considering the results of studies on healthy brains in combination with the effects of lesions in patients is it possible to make strong inferences about the functional specialization of regions within the human parietal cortex.

## Box 2 Questions for future research

• Just as novel parietal regions might have emerged in humans, might there also be specializations in monkey parietal regions that are not present in man (e.g. related to the special visuomotor abilities that might be required for an arboreal existence)?
• What are the differences in underlying cognitive deficits between neglect following left- and right-hemisphere lesions? For example, the data reviewed here would suggest that major deficits in sustained attention would not be expected following left-IPL lesions. Better characterisation of neglect following left-hemisphere lesions would be an important step forward.
• Although several dissociable behavioural components of neglect have now been clearly identified, their corresponding anatomical loci have proved challenging to establish. Component deficits might be masked or confounded by interactions between different components. Similarly, a faithful analysis of the underlying anatomy might require lesion-mapping methods that capture correlations between different anatomical areas. Can such richly multivariate analyses be successfully performed with the necessarily constrained datasets used in human lesion-mapping research?
• The fine functional parcellation of the cortex achieved with fMRI is yet to be matched by a correspondingly detailed map of the connections between remote areas. Therefore, the behavioural effects of white-matter lesions are hard to relate to the underlying functional architecture. Would the development of a detailed standardized map of white-matter tracts improve our understanding of the consequences of damage that involves parietal grey- and white-matter regions?
